# Identification of novel molecular determinants of co-receptor usage in HIV-1 subtype F V3 envelope sequences

**DOI:** 10.1038/s41598-020-69408-x

**Published:** 2020-07-28

**Authors:** Maximiliano Distefano, Esteban Lanzarotti, María Florencia Fernández, Andrea Mangano, Marcelo Martí, Paula Aulicino

**Affiliations:** 1 Laboratorio de Biología Celular Y Retrovirus- CONICET, Hospital de Pediatría “J.P. Garrahan”, Ciudad Autónoma de Buenos Aires, Argentina; 2 Departamento de Computación, Facultad de Ciencias Exactas Y Naturales, Universidad de Buenos Aires, Ciudad Autónoma de Buenos Aires, Argentina; 3 Departamento de Química Biológica, Facultad de Ciencias Exactas Y Naturales, Universidad de Buenos Aires, Ciudad Universitaria, Ciudad Autónoma de Buenos Aires, Argentina

**Keywords:** Protein sequence analyses, Infection

## Abstract

HIV-1 determinants of coreceptor usage within the gp120 V3 loop have been broadly studied over the past years. This information has led to the development of state-of the-art bioinformatic tools that are useful to predict co-receptor usage based on the V3 loop sequence mainly of subtypes B, C and A. However, these methods show a poor performance for subtype F V3 loops, which are found in an increasing number of HIV-1 strains worldwide. In the present work we investigated determinants of viral tropisms in the understudied subtype F by looking at genotypic and structural information of coreceptor:V3 loop interactions in a novel group of 40 subtype F V3 loops obtained from HIV-1 strains phenotypically characterized either as syncytium inducing or non-syncytium inducing by the MT-2 assay. We provide novel information about estimated interactions energies between a set of V3 loops with known tropism in subtype F, that allowed us to improve predictions of the coreceptor usage for this subtype. Understanding genetic and structural features underlying HIV coreceptor usage across different subtypes is relevant for the rational design of preventive and therapeutic strategies aimed at limiting the HIV-1 epidemic worldwide.

## Introduction

Human immunodeficiency virus type 1 (HIV-1) enter its target cell through the interaction of the viral glycoprotein gp120, coded in the envelope gene, initially with the cellular receptor CD4^1^ and subsequently with CCR5 or CXCR4, the two main coreceptors. The CD4-gp120 contact induces a conformational change in gp120 that exposes the third variable loop (V3) and allows its interaction with the coreceptor leading to viral entry to the cell^2,3^. HIV-1 coreceptor preference for either CCR5 or CXCR4 is known as viral tropism. Thus, viruses that use CCR5 are termed R5-using and those that use CXCR4 are termed X4-using. Those viruses capable of infecting the CD4 T-cell through any of these coreceptors are termed X4R5-using.

HIV-1 tropism can be determined by phenotypic assays that evaluate the ability of the viral isolates to use the different coreceptors in vitro. The most traditional phenotypic assay -used since early in the HIV epidemic to define tropism in clinical specimens-relies on the ability of HIV-1 to induce syncytia formation upon infection of the immortalized lymphoid cell line MT-2, that expresses CD4 and the CXCR4 coreceptor^4^. Thus, X4 or X4R5-using viruses can infect MT-2 cells and are termed syncytium inducing (SI), while R5-using viruses cannot infect MT-2 cells and are termed non-syncytium inducing (NSI).

The presence of SI HIV-1 strains has been associated to an accelerated AIDS progression, immunosuppression^5^ and lack of response to antiretroviral therapy with the CCR5 antagonist Maraviroc^6^. Determinants that govern coreceptor usage have been mapped to the envelope gene, especially to the variable regions, and in particular the gp120 V3 loop. This has led to the development of multiple rules and algorithms that allow prediction of coreceptor usage based on V3 sequence information. The simplest rules are those known as 11/25 or 11/24/25. These rules predict X4-using strains in the presence of positively charged residues at positions 11, (24) and/or 25. Despite these rules have shown high specificity, their specificity even for subtype B is low^7^. Other methods assess the entire V3 sequence and assign the viral tropism based on algorithms trained with sequences with known phenotype. The most popular ones are Geno2Pheno (G2P)^8^ and WebPSSM^9^. While valuable, most of these machine-learning genotypic coreceptor tropism testing (GTT) tools have been developed primarily for HIV-1 subtype B, the most prevalent HIV-1 subtype in Europe and the US and therefore the most widely studied. However, the performance of GTT methods for predicting HIV-1 tropism is variable between B and non-B subtypes^10,11,12^, and particularly poor for subtype F strains^13^. Improved predictions for several non-B subtypes were obtained by using subtype-specific training sets for the development of the GTT tools (e.g. PhenoSeq on A/CRF-02 AG, C, D and CRF_01 AE^14^, WebPSSM-C on subtype C^15^, THETA^16^ on CRF_02 AG and SCOTCH on subtype A^17^, but none of them included subtype F.

Originally found in Central Africa, HIV-1 subtype F represents a low proportion (approximately 1%) of the total circulating strains worldwide, and its circulation is restricted to a few countries—namely Congo Democratic Republic, Romania, Spain and Brazil. However, subtype F has gained epidemiological importance in South America due to the emergence and spread of different BF recombinant strains whose frequency increase from north to south at the expense of a reduction in HIV-1 infections caused by subtype B. While more than 50% of HIV-1 infections in Latin American countries are caused by different BF recombinants, there is still very limited information on the biological properties associated to these subtypes. Importantly, of the 14 BF Circulating recombinant forms (CRF_BFs) identified in Brazil, Uruguay, Chile and Argentina, 9 (64%) are subtype F in the gp120 V3 loop region (https://www.hiv.lanl.gov/content/sequence/HIV/CRFs/CRFs.html).

Using molecular dynamics (MD) simulations and free energy calculations, Tamamis and Floudas described for the first time in 2013–2014 complete complex structures of gp120 V3 loop in association with CXCR4^18^ or CCR5^19^. This allowed the identification of new genetic and structural features underlying HIV-1 coreceptor usage^20^, and also the development of new tropism prediction tools^21^. However, the extent with which protein–protein interactions found in subtype B V3 loop sequences are conserved across different subtypes is unknown and merits further research.

In the present work we sought to identify putative determinants of viral tropism and subtype-specific molecular interactions relevant for the V3:coreceptor binding using a novel dataset of subtype F V3 loop sequences obtained from BF recombinant HIV-1 strains with known SI/NSI phenotype.

## Materials and methods

### Study population

Whole blood samples (5–10 ml) were obtained by venipuncture into EDTA-containing tubes from HIV-1 infected children or adolescents attending Hospital de Pediatría "J. P. Garrahan" in Buenos Aires Argentina from 1986 to December 2015. Peripheral Blood Mononuclear Cells (PBMCs) were separated by Ficoll-Hypaque density gradient centrifugation under sterile conditions, two million PBMC aliquots were stored at -20 °C for PCR amplification of the V3 loop HIV-1 *env* fragment, and the remaining PBMCs were cultured for in vitro isolation of HIV-1. The study was reviewed and approved by the Garrahan Hospital Ethics Committee (IRB00004240) before it began. Informed consent was obtained from the children´s parents or legal guardians in all cases. All methods were performed in accordance with the relevant guidelines and regulations.

### In vitro characterization of SI/NSI phenotype by MT-2 assay

HIV-1 was isolated by cocultivation of cells as previously described by the AIDS Clinical Trials Group^22^. Briefly, PBMCs from both the patient and HIV-1-seronegative blood donors pre-stimulated for 24–72 h with 5 ug/ml of phytohemagglutinin (PHA) (Difco Laboratories) were cocultured at a final concentration of 2 × 10^6^ cells/ml. Cocultures were maintained for 28 days in RPMI 1640 medium (Gibco BRL, Invitrogen) supplemented with 20% heat inactivated fetal bovine serum (FBS), 5 U/ml interleukin 2 (IL-2) (Sigma Aldrich), and 10 ug/ml gentamicin (Gibco BRL Invitrogen). Measurement of HIV-1 p24 Ag of coculture supernatants was performed with a commercial assay kit (Vironostika HIV-1 Antigen, BioMerieux). For phenotype characterization of SI or NSI, HIV-1 culture supernatants were tested on MT-2 cells following the protocol by Japour et al^23^, and as previously described by our group^24^, using positive and negative controls.

### Amplification and sequencing of HIV-1 C2-V5 *env* segments

Two million PBMCs were treated with a lysis buffer containing Proteinase K and stored at − 20 °C for subsequent PCR amplification of a 372 bp C2-V5 HIV-1 *env* gene fragment comprising the V3 region (positions 7,001 to 7,339 relative to the HXB2; GenBank accession number K03455) with primers JA10/JA11 using PCR conditions previously described^25^. The PCR products were purified with QIAquick purification columns (QIAGEN, Germany), and then sequenced using the DYEnamic ET Terminator Cycle sequencing kit v1.1 (Amersham Biosciences, England). Sequencing reactions were run on an ABI 3500 automated sequencer and analyzed with the Variant Reporter Software 2 (Applied Biosystems, USA). V3 loop sequences were identified within the HIV-1 C2-V5 *env* fragment. Amino acid V3 loop sequences are available as Supplementary Information.

### Analysis of HIV-1 V3 loop sequences

Amino acid composition of V3 loop sequences were analyzed by WebLogo^26^ (available at: https://weblogo.berkeley.edu/). Amino acid V3 loop variability was evaluated by calculating Shannon’s entropy using a Monte Carlo randomization strategy available at Los Alamos Entropy webservice (https://www.hiv.lanl.gov/content/sequence/ENTROPY/entropy.html). For prediction of coreceptor usage, V3 loop sequences were submitted to two web servers: (1) Geno2pheno web tool (https://coreceptor.geno2pheno.org/) setting FPR to 10% and (2) WebPSSM_sinsi_ (https://indra.mullins.microbiol.washington.edu/webpssm/).

### Phylogenetic analysis of HIV-1 env segments

After visual inspection and manual correction, HIV-1 V3 loop env sequences were aligned with Los Alamos HIV-1 group M subtype reference sequences using Clustal X program^27^. For subtype assignment, a Neighbor-Joining phylogenetic tree was built in MEGA 5.0 program^28^, using full-length subtype reference genome sequences A–C and F–H and J retrieved from Genbank. Bootstrap method was used to assess the stability of the nodes.

### Reference subtype B V3 loop dataset

A reference dataset including only subtype B V3 loop sequences of 35 amino acids in length, and with well characterized viral tropism was obtained from the curated V3 loop dataset compiled by Kieslich^21^. One hundred and two V3 loop sequences were randomly selected (42 with X4 tropism, 60 with R5 tropism).

### V3loop:CCR5 and V3loop:CXCR4 model construction

Models of V3 loop were produced in complex with both coreceptors, CCR5 and CXCR4. Template based homology modeling was used to make structures of subtype F and subtype B loops interacting with each coreceptor, based on model structures proposed by Tamamis and Floudas, of a dual tropic V3 loop in complex with CCR5^19^ and CXCR4^18^. Since 10 template structures were available for each coreceptor, MODELLER’s loop optimization routine was used to produce 10 new models for each template obtaining a final count of 100 models per V3 loop of F subtype.

### Interaction energy calculation

We estimated the contribution to CXCR4 interaction energy of each V3 loop residue using a residue-residue coarse grain potential based on contacts deduced from distances between each V3 loop and coreceptor residues^29^. We defined contacts in the interface between each loop and coreceptor, as any pair of residues having their alpha carbons at less than a fixed distance depending on the type of interacting residues. Average interaction energy was estimated for each position separately adding contacts interaction energy found in 100 models and dividing it by 100. Python scripts used for modelling and energy calculation can be found at https://github.com/mxdistefano/V3-X4-energy.

## Results

### Comparison between subtype F and subtype B V3 loop sequences from X4-using and R5-using viruses

Forty V3 loop sequences were obtained from 37 HIV-1 infected children and adolescents. All sequences clustered with subtype F1 reference sequences in phylogenetic trees (Supplementary Figure 1). In 24 cases, viral isolates showed formation of syncytia upon infection of the MT-2 cell line, being characterized as SI-F or X4-F strains. Another 16 did not show evidence of syncytia and therefore were characterized as NSI-F or R5-F strains. In 3 individuals, V3 loop sequences were obtained at two different time points.

**Figure 1:**
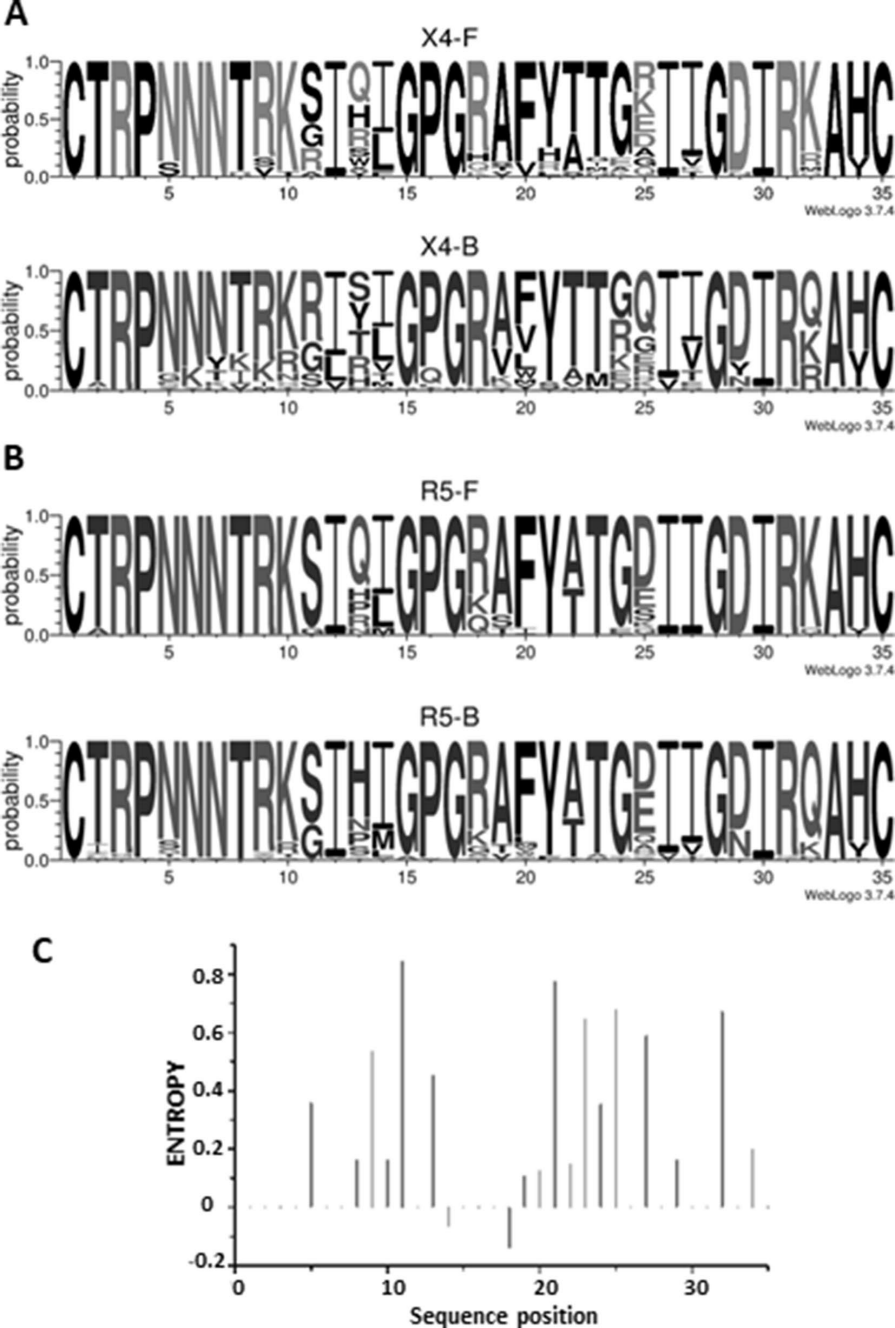
Sequence logos of HIV-1 subtype F and B V3 loop data subsets. (**A**) X4-F and X4-B, (**B**) R5-F and R5-B. (**C**) Difference in entropy calculations between X4-F and R5-F data set using a Monte Carlo randomization test with 1,000 iterations. Black bars indicate statistically significant positions.

Subtype F V3 loop sequences were submitted to Geno2pheno (FPR = 10%) and WebPSSM_sinsi_ online prediction tools. Of the 24 X4-F strains, only 13 (54%) were correctly classified as X4-using by WebPSSM_sinsi_ and 14 (58%) by Geno2Pheno. For R5-F V3 loops, 15 of 16 (94%) sequences were correctly classified as R5-using, both by Geno2Pheno and WebPSSM_sinsi_. Due to the low sensitivity detected, sequence composition and variability were compared along the 35 amino acid positions of the V3 loop in each of the subtype F datasets, using sequence logos and Shannon’s entropy (Fig. 1C). Visual inspection of the logos revealed important differences between the X4-F and R5-F datasets at positions 11 and 25, known to be associated to HIV-1 tropism. Interestingly, S was the most frequent amino acid at position 11 both in X4- and R5- subtype F using strains, although X4-F also showed G or R amino acids at this position. At position 25, D predominated in R5-F, whereas both negatively (D and E) and positively charged amino acids (R and K) were found at equal frequency in the X4-F dataset. Sequence entropies were compared between the X4-F and R5-F datasets (Fig. 1C). Results show a higher diversity for X4-F at 3 positions within the V3 loop (11, 21 and 25). At position 21, R5-F carried exclusively Y, while other amino acids (H, R or L) were also found in X4-F strains.

In order to identify differences associated to HIV-1 subtype in sequences belonging to X4-using or R5-using viruses, we compared our subtype F datasets to the reference dataset X4-B (Fig. 1A) and R5-B (Fig. 1B). Interestingly, subtype-specific differences in the predominant amino acids were observed at positions 11 and 25 only for the X4-using viruses (X4-F vs X4-B). At position 11, S was present in 54% of the sequences for subtype F, followed by G and R at equal frequencies (20%). In the X4-B dataset, the most frequent amino acid at position 11 was R. This was present in more than 70% of the sequences, followed by G and S (20% and 10%, respectively). At position 25, E, R, K and D represented 12% to 25% of the amino acid diversity in subtype F. In the subtype B dataset, Q was the most frequent amino acid (> 50%), followed by G, E, R, D, K and N in minority proportions. In the case of the viruses that use only the CCR5 coreceptor (R5-F vs R5-B), the amino acid composition was conserved between the two subtypes.

### Comparison of V3:CXCR4 interaction energies between subtypes B and F

Since interaction of V3 with the cellular CXCR4 or CCR5 co-receptors involves distinct amino-acid contacts, we hypothesize that similar V3 residues in X4 and R5 strains may have different impact when binding with each co-receptor. In order to test this hypothesis, we studied the interaction between V3 and each coreceptor using homology modelling. A detailed analysis of the interactions found for X4 V3 loop sequences as well as the amino acid frequency at each position is shown in Supplementary Table 1. Ninety-two residue-residue contacts were found for X4-F:CXCR4 and 85 for R5-F:CCR5. The same analysis performed in our reference subtype B datasets showed 142 residue-residue contacts for X4-B:CXCR4 and 157 for R5-B:CCR5.

**Figure 2:**
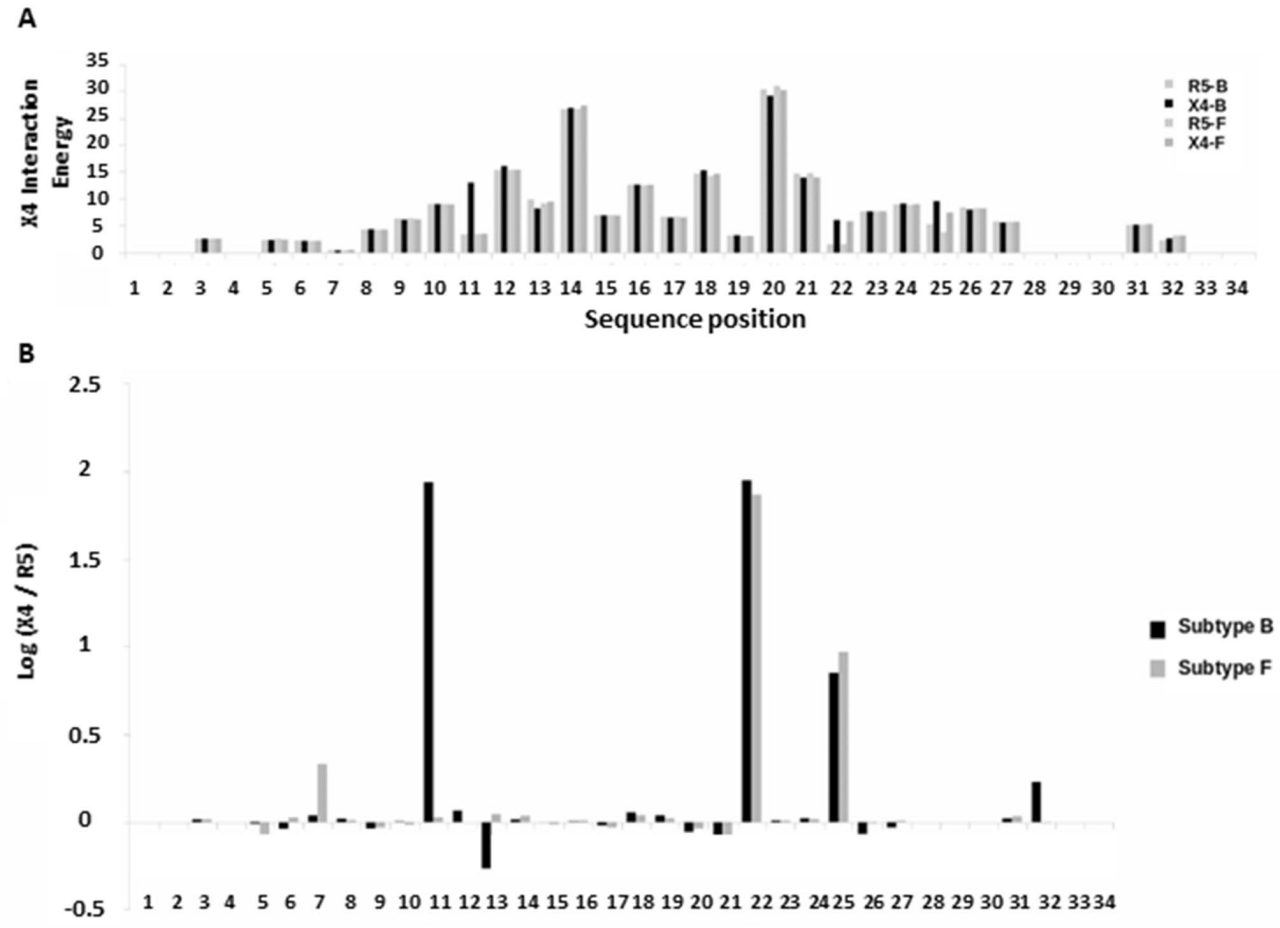
Contact energy comparison between X4 and R5 V3 loop positions with CXCR4 coreceptor. (**A**) Interaction energy is estimated for each position separately (see methods) and median of all models is calculated for X4-F, R5-F, X4-B and R5-B. (**B**) Median interaction energies at all positions are compared using log odd ratio between X4 tropism and R5 tropism for both subtypes B and F.

Comparison of residue-residue contacts between subtypes in X4-using viruses showed that a set of coreceptors residues involved in subtype B were absent in subtype F, mostly due to the absence of V3 loop interacting side chains in the latter. Among the V3:CXCR4 interactions involving amino acids present in both subtypes at the same position, differences were found in 8 contacts. Of them, 7 were only present in the X4-B dataset (I:14-D:187, R:18-Y:116, A:19-R:188, V:19-H:281, K:24-K:25, K:24-C:28, R:25-M:1) and 1 (K:25-K:25) was only present in the X4-F dataset. Interactions between V3:CCR5 showed differences between subtypes at 4 positions. In all cases, these were present in the R5-B and absent in R5-F datasets (N:7-Y:14, H:13-S:179, R:18-E:283 and Q:32-D:11). The median contact energy per V3 position was compared between X4:CXCR4 and R5:CXCR4 to quantify differences in the binding of each viral subtype. Most of X4 and R5 contacts with CXCR4 were observed between V3 position 8 and 27 for both subtypes. As shown in Fig. 2A, in all viral datasets, V3 positions with highest interaction energy (more than the median = 6.5 of all positions) were 12, 13, 14, 15, 16, 17, 18, 20, 21, 23, 24 and 26. Surprisingly, rule position residues (11 and 25) did not have high energy contacts, except in the case of X4-B for position 11. Moreover, we compared tropism defining interactions using log odd ratios between the computed interaction energy for each receptor at all positions, and detected that position 11 has strong selectivity, only for subtype B. Additionally, large interaction energy differences were observed at positions 22 and 25 in both viral datasets. These two positions would be the most discriminant between both tropisms (Fig. 2B).

### Discrimination of CXCR4 and CCR5 using sequences for subtype F

Due to the differences observed in energy contributions at positions 11, 22 and 25 between subtypes B and F, we tested whether these could be used to improve prediction of HIV-1 tropism for our dataset of subtype F V3 loop sequences.

Given a V3 sequence, we used MODELLER to produce 100 model structures of the loop in complex with CXCR4. We estimated interaction energy for each loop position separately searching for contacts between V3 loop and CXCR4 residues (for details see methods). Next, we averaged contacts energy for all 100 models to finally obtain a score for each position and summed up only those obtained for positions 11, 22 and 25 (11,22,25 X4 Energy). Additionally, we combined this energy-based method with Geno2pheno in a mixed model using a simple linear model (i.e.: W * Geno2pheno + (1 − W) * 11–22–25 X4 Energy) with a weight W = 0.3 (Mixed model W = 0.3). We screened for this weight in the subtype B dataset which is independent of subtype F dataset.

**Figure 3:**
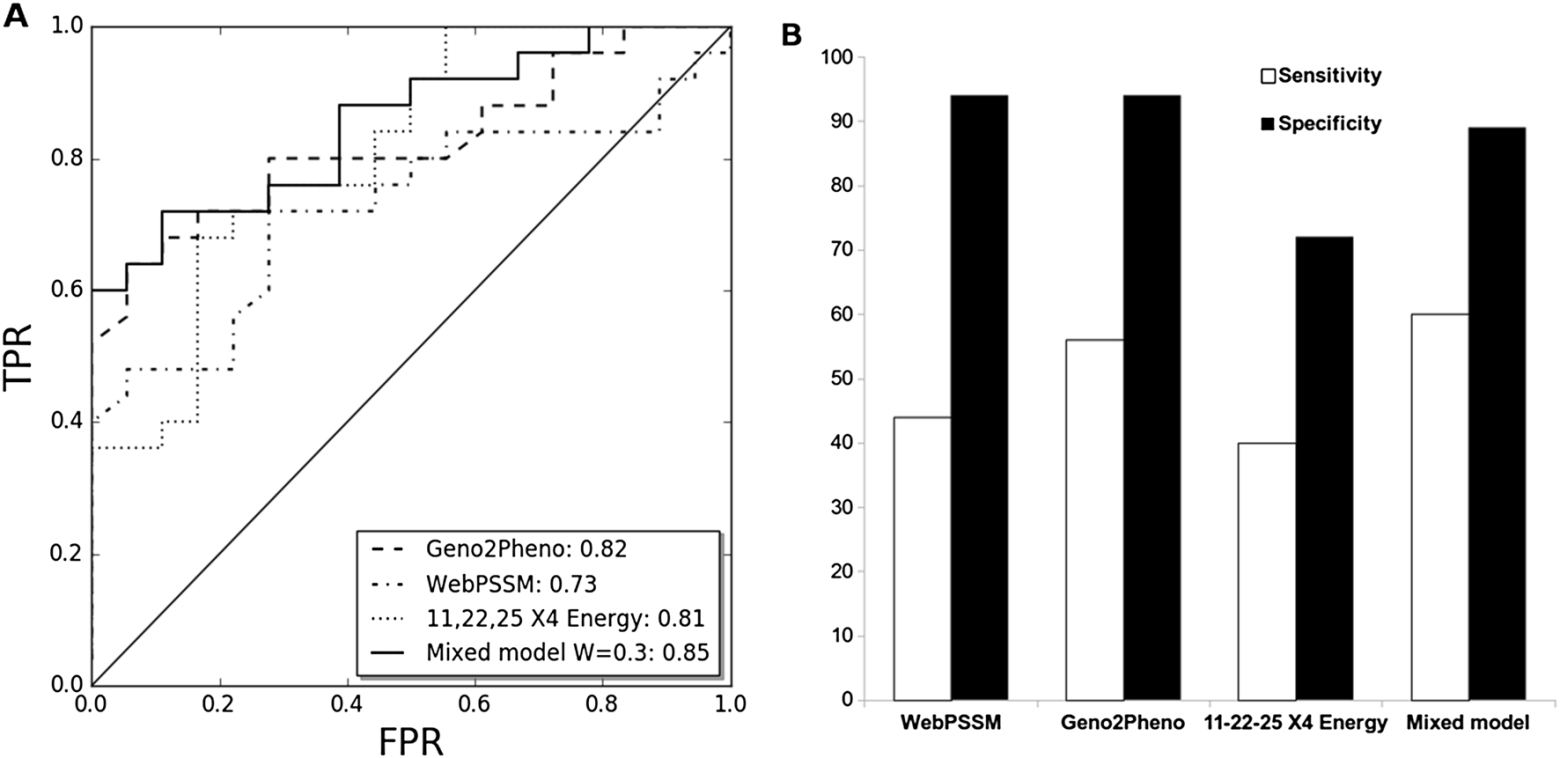
Discrimination between X4 and R5 using V3 loops. (**A**) ROC curves for viral tropism prediction in subtype F dataset. (**B**) Sensitivity and specificity of available tools compared to our method based on structure and a mixed model combining it with Geno2Pheno using a linear combination (W = 0.3). *TPR* true positive rate, *FPR* false positive rate.

The performance of each method was compared through the area under the receiver operating characteristic curves (AUC values of the ROC curve) obtained for each method’s raw scores (Fig. 3A). The Mixed model W = 0.3 was the best performing method (AUC = 0.85). Geno2pheno performed second (AUC = 0.82), 11,22,25 X4 Energy performed third (AUC = 0.80) and WebPSSM performed fourth (AUC = 0.73). It is worth noting that the high performance of the 11,22,25 X4 Energy method was not expected since it only takes into account the contact energy of specific amino acids at each position, and not the amino acid type.

Finally, we made predictions with each method setting as false positive rate cutoff 10%, 15% and 20% by using cross-validation. Subtype F dataset was divided into 5 partitions, preserving the proportion V3 loops from X4-F and R5-F viruses (stratified fivefold). For each partition, we looked for the score cutoff that gives the selected false positive rate using the other 4 partitions and assigned tropism using this cutoff. The sensitivity of the mixed model was slightly higher at FPRs of 15% and 20% (68%) than at FPR of 10% (60%), while the specificity remained equal for all the FPRs tested (89%) (Supplementary table 3). Since the European Consensus group on clinical management of HIV-1 tropism testing recommends the use of FPR = 10%, comparison of specificity and sensitivity of our method against Geno2Pheno and PSSM was done using this threshold (Fig. 3B). The 11,22,25 X4 Energy method showed the lowest sensitivity and specificity. However, the Mixed model W = 0.3 obtained an improved sensitivity due to more X4-using V3 loops classified correctly (60% for mixed model vs 56% for Geno2Pheno and 44% for WebPSSM) but with a lower specificity (89% for mixed model vs 94% for Geno2Pheno and WebPSSM).

## Discussion

Determinants of HIV-1 coreceptor usage have been widely studied in subtype B viruses. However, information is scarce or missing for less prevalent subtypes such as subtype F. By using HIV-1 V3 loop V3 structures defined by Tamammis et al^18,19^, we modelled for the first time a set of V3 loop subtype F sequences obtained from SI (X4-using) and NSI (R5-using) viruses. An in-depth analysis of molecular interactions occurring in silico between each of the two main cellular coreceptors used for HIV-1 binding (CXCR4 and CCR5), and V3 loop sequences revealed subtype-specific differences associated with HIV-1 coreceptor usage. Combination of the Geno2Pheno GTT tool and interaction energies at specific positions within the V3 loop -namely 11, 22, and 25-in a mixed model resulted in an improved method for prediction of HIV-1 tropism of subtype F strains.

In subtype B HIV-1 strains, sequence information contained in the gp120 V3 loop is usually enough for the correct characterization of HIV-1 coreceptor usage with different GTT tools. However, an evaluation of concordance between the results obtained using GTT tools -taking the phenotypic results as a reference- revealed differences in their performances, with WebPSSM_X4R5_ (91.4%), WebPSSM_sinsi_ (88.6%), and geno2pheno (88.6%) being the most accurate for subtype B, and WebPSSM_sinsi_ (83.8%) being the most accurate for non-B strains^14^. Indeed, fine-tuning or subtype-specific rules have been reported to compensate for these inadequacies in several non-B subtypes, such as subtype C^15^, A/ CRF02_AG^14,16,30^, subtype D^31^, and CRF01_AE^32^. Development of these methods is largely dependent on the dataset used for training. Importantly, only 36 (1.1%) of the 3,334 V3 loop sequences with known phenotype available in GenBank, belong to subtype F or contain subtype F genomic segments (24 BF1 from Argentina, Brazil and Spain, 5 F1 from Spain, Italy, and Russia, 4 F2 from Cameroon and Russia, 2 CRF47_BF from Spain, 1 DF1G from Spain). Of them, 9 are SI and 27 NSI. This clearly contrasts the overrepresentation of subtype B V3 loop sequences in public databases (1,868, 56%), and at the same time highlight the need to increase the number of subtype F V3 loops from HIV-1 strains with known phenotype.

As previously reported, we observed that V3 loop sequences from subtype B X4-using strains usually follow the 11/24/25 rule, in particular at position 11. In contrast, subtype F V3 loops rarely showed positively charged AA (K or R) at positions 11 and 24 (R at position 11 in 5/24 (21%), K at position 24 in 1/24 (4%) of X4-using sequences). These results are in agreement with previous studies that show that determinants of MT-2 tropism in subtype F strains are not only dependent of positively charged AA at positions 11 and 25^33^. In fact, it has been proposed that V3 loops that do not contain positively charged AA at position 11 could also use CXCR4 co-receptor because Tyr12 could be also attracted to positive charged residues at position 9 and 10, stabilized by residue 10^14^. In our datasets Tyr12 interacts with all AA at position 9 in both subtype B and F V3 loops from X4-using strains, supporting its role in the stabilization. However, R5-using strains also interacted with CXCR4 with comparable energy thus limiting its importance in conditioning coreceptor usage. In subtype F, positions 22 and 25 were the strongest feature descriptors of HIV coreceptor usage due to their differences in CXCR4 binding energy contribution. Noteworthy, these differences are not only attributable to the nature of the residues at positions 22 and 25, but are a consequence of the AA composition of the V3 loops that affect its conformations, and therefore the contacts established to the coreceptor.

Previously, structural data from molecular modeling of V3 with or without interaction with the CCR5 and CXCR4 coreceptors was used for identification of molecular determinants of HIV-1 coreceptor usage in V3 loop. While some studies evaluated the interaction between gp120 and CCR5 N terminus^34^, others used only V3 conformation to identify important features—namely hydrogen-bond donor sites and aliphatic side chains^35^. Recently, Kieslich et al. identified interacting residue pairs between the V3 loop and CCR5 or CXCR4 coreceptors by using molecular dynamics simulations on a large dataset including 2,455 V3-loop sequences of various lengths and subtypes (B, C, A, A/G, A/E)^21^. The authors described 18 top-selected interactions. In our study, we found that 4/10 were confirmed for subtype B and 2/10 for subtype F in X4-using V3 loops. For R5-using V3 loops, 5/8 interactions were confirmed for subtype B and 4/8 for subtype F. Also, subtype F V3 loops showed a lower number of contacts with both CCR5 and CXCR4 coreceptors in comparison to subtype B from our dataset, even after controlling for differences attributable to amino acid diversity. Altogether, these results suggest the presence of subtype-specific interactions not previously described.

The addition of a novel energy-based classifier algorithm using subtype F to existing bioinformatic tools improved the sensitivity and specificity of detection of CXCR4 using virus, but still some sequences remained misclassified. This could be explained by the presence of dual tropic X4/R5 strains that share certain V3 loop characteristics with X4- and others with R5-using variants as previously found in subtype D viruses^36^. However, characterization of dual tropic HIV-1 viruses in F or BF subtypes has not yet been described. Since our sequences were obtained from bulk PBMC without cloning, R5-using V3 loops in SI datasets could also be explained by the presence of minority X4-using variants (< 20%) not detectable by Sanger sequencing. The lack of experimental confirmation of genotype–phenotype correlations of particular V3 sequences could be a limitation of our study. However, previous studies have shown that bulk sequencing is a valuable tool for predicting HIV-1 tropism due to the good correlation between direct sequencing and clonal analysis of the V3 loop^37^. Another limitation of the study is the low number of sequences used for training of the predictive methods which affect the accuracy in a direct manner. Despite the limitations, this method could provide an improved classification of V3 loop sequences in comparison to the available GTT tools for BF recombinant HIV-1 strains circulating in Latin America.

Overall, we provide relevant information about how subtype F V3 loop interacts with the co-receptors CCR5 and CXCR4. Observed differences allow to understand the low sensitivity of the subtype B-based GTT prediction methods for subtype F sequences. Importantly, information obtained from molecular interactions proved to be useful for improving prediction of co-receptor usage. Other limitations of the study are the low number of sequences used for training of the predictive methods, which affect the accuracy in a direct manner; and the fact that training was done with V3 sequences from viral isolates from only one country. Despite the limitations, our method could provide an improved classification of V3 loop sequences in comparison to the available GTT tools not only for BF recombinant HIV-1 strains circulating in Latin America, but also for pure F1 or other recombinant HIV-1 strains with F1 env genomic fragments, such as CRF05_DF. While our method was trained with sequences from children and adolescents, we do not expect this to condition its use in other populations. Understanding the impact of HIV-1 inter-subtype diversity on the interactions between gp120 V3 loop and CXCR4 or CCR5 are of importance for the design of preventive and therapeutic strategies aimed at limiting the HIV-1 epidemic worldwide.

## Supplementary information

Supplementary Information.
