# Supplementary material for: Identification of novel molecular determinants of co-receptor usage in HIV-1 subtype F V3 envelope sequences
Source: Sci Rep. 2020 Jul 28;10:12583. doi: 10.1038/s41598-020-69408-x (PMC7387458; doi:10.1038/s41598-020-69408-x)
Supplement: Supplementary file 1 — Supplementary Information. [file 41598_2020_69408_MOESM1_ESM.pdf]

Identification of novel molecular determinants of co-receptor usage in HIV-1 subtype F V3 envelope sequences

Maximiliano Distefano, Esteban Lanzarotti, María Florencia Fernández, Andrea Mangano, Marcelo Martí, Paula Aulicino

Supplementary table 1: Comparative table of amino acid frequency and contacts between X4-F and X4-B with CXCR4 (A) and R5-F and R5-B with CCR5 (B).

A

	1	2	3	4	5	6	7	8	9	10	11	12	13	14	15	16	17	18	19	20	21	22	23	24	25	26	27	28	29	30	31	32	33	34	35
Subtype F V3 loop aminoacid frequency	C (100)	T (100)	R (100)	P (100)	N (88.46)<br>S (11.54)	N (100)	N (100)	R (3.85)<br>T (96.15)	R (84.62)<br>S (7.69)<br>V (7.69)	K (96.15)<br>T (3.85)	A (3.85)<br>G (19.23)<br>R (19.23)<br>S (57.69)	I (100)	A (3.85)<br>H (19.23)<br>Q (42.31)<br>R (15.38)<br>S (7.69)<br>W (7.69)<br>Y (3.85)	I (65.38)<br>L (30.77)<br>F (3.85)	G (100)	P (100)	G (100)	R (80.77)<br>Q (7.69)<br>H (7.69)<br>K (3.85)	A (88.46)<br>S (3.85)<br>T (3.85)<br>V (3.85)	F (88.46)<br>V (11.54)	H (11.54)<br>L (3.85)<br>R (7.69)<br>Y (76.92)	A (38.46)<br>S (3.85)<br>T (57.69)	G (3.85)<br>I (3.85)<br>M (3.85)<br>N (3.85)<br>T (84.62)	A (3.85)<br>E (7.69)<br>G (84.62)<br>K (3.85)	A (7.69)<br>D (11.54)<br>E (23.08)<br>G (7.69)<br>K (19.23)<br>Q (7.69)<br>R (23.08)	I (100)	I (84.62)<br>K (3.85)<br>T (3.85)<br>V (7.69)	G (100)	D (96.15)<br>N (3.85)	I (100)	R (100)	K (80.77)<br>M (3.85)<br>Q (3.85)<br>R (11.54)	A (100)	H (84.62)<br>Y (15.38)	C (100)
subtype F V3 loop-CXCR4 interactions			R - D22			N - S23		T/R - Y21<br>R - K25	R/V/S - Y12	T-Y:21<br>K/T - D193<br>K/T - L266	R - M1<br>R - Y7<br>R - Y12	I - F189<br>I - Y190	R/Q/H/S/W/Y- M1<br>W/Y - E2<br>R/H/Q - R30	F - E2<br>I/L- S178<br>F - A180<br>I/L/F - I185<br>F/L - D187		P- F36<br>P- L41	G - W94	H/K - W94<br>R/H/K - H113<br>R/H/K/Q - R188	S - H281<br>S/T/V - I284	F/V - R188<br>F/V - Y190<br>F/V - V196<br>F - Q200<br>F - I284	R/Y- R30<br>R/H/Y/L- H281	T - L266	M/T/I - M1<br>M - C28<br>M/T/I - F29	A- K25<br>K- P27	**K**/A- K25<br>R/Q/K - P27	I - K25<br>I - S23<br>I - P27					R - E14<br>R - G17<br>R - S18	R - G17		Y- D20	
	1	2	3	4	5	6	7	8	9	10	11	12	13	14	15	16	17	18	19	20	21	22	23	24	25	26	27	28	29	30	31	32	33	34	35
Subtype B-X4 V3 Loop aminoacid frequency	C (100)	T (92.86)<br>A (4.76)<br>I (2.38)	R (100)	P (100)	N (85.72)<br>G (7.14)<br>S (2.38)<br>Y (2.38)<br>F (2.38)	N (80.95)<br>K (16.67)<br>Y (2.38)	N (71.43)<br>Y (11.9)<br>T (9.52)<br>K (4.76)<br>H (2.38)	T (69.05)<br>K (14.29)<br>I (11.9)<br>V (4.76)	R (76.19)<br>K (16.67)<br>I (4.76)<br>M (2.38)	K (66.67)<br>R (19.05)<br>N (7.14)<br>Q (2.38)<br>G (2.38)<br>T (2.38)	R (59.52)<br>G (26.19)<br>S (11.9)<br>K (2.38)	I (71.43)<br>L (21.43)<br>V (7.14)	S (26.19)<br>Y (23.81)<br>T (21.43)<br>R (16.67)<br>H (9.52)<br>P (2.38)	I (47.62)<br>L (28.57)<br>V (9.52)<br>T (7.14)<br>M (4.76)<br>A (2.38)	G (97.62)<br>S (2.38)	P (80.95)<br>Q (14.29)<br>T (2.38)<br>L (2.38)	G (97.62)<br>S (2.38)	R (97.62)<br>K (2.38)	A (64.29)<br>V (23.81)<br>K (7.14)<br>Y (2.38)<br>R (2.38)	F (45.24)<br>V (23.81)<br>L (14.29)<br>W (7.14)<br>Y (4.76)<br>M (4.76)	Y (92.86)<br>S (7.14)	T (80.95)<br>A (11.9)<br>V (4.76)<br>R (2.38)	T (85.71)<br>M (11.9)<br>L (2.38)	G (40.48)<br>R (28.57)<br>K (14.29)<br>E (9.52)<br>D (7.14)	Q (54.76)<br>G (14.29)<br>R (9.52)<br>E (9.52)<br>D (4.76)<br>K (4.76)<br>N (2.38)	I (90.48)<br>V (9.52)	I (59.52)<br>V (23.81)<br>T (9.52)<br>E (4.76)<br>R (2.38)	G (100)	D (76.19)<br>Y (11.9)<br>N (9.52)<br>T (2.38)	I (97.62)<br>T (2.38)	R (97.62)<br>K (2.38)	Q (50)<br>K (28.57)<br>R (21.43)	A (97.62)<br>T (2.38)	H (66.67)<br>Y (30.95)<br>R (2.38)	C (100)
Subtype B V3 loop-CXCR4 interactions			R - D22		F/Y - D20<br>Y- D22	N/K/Y - S23		I/K/T/V - Y21<br>I/K/V - K25	R/I/M - Y12	R - S18<br>R/T - Y21<br>R/N/Q/K/T - D193<br>R/T/K - L266	R/K - M1<br>R - Y7<br>R - Y12	I/L/V - F189<br>I/L/V - Y190	R/H/P/S/T/Y - M1<br>Y- E2<br>R/H - R30	I/L/M - S178<br>M - R183<br>I/L/M/T/V - I185<br>**I**/L/M - D187<br>M - F189	S - I185	Q/L/P/T - F36<br>T- N37<br>Q/L/P/T - L41<br>L- S285	G/S - W94	K - W94<br>K/R - H113<br>**R** - Y116<br>K/R - R188	**A**/K - R188<br>R/**V** - H281<br>R/K/Y/V - I284<br>R/K/Y- S285	L/M/F/Y/V - R188<br>L/M/F/V/W - Y190<br>L/M/F/W/Y/V - V196<br>F - Q200<br>W - D262<br>F - I284	Y - R30<br>S/Y - H281	R/T/V - L266	L/M/T - M1<br>L/M - C28<br>L/M/T - F29	R/**K** - K25<br>K - P27<br>R/**K** - C28	**R** - M1<br>K/Q/R - P27	I - S23<br>I/V - K25<br>I - P27					R - E14<br>R - G17<br>R - S18	R - G17		Y- D20	

Shaded amino acids did not show contacts. Amino acids that were found to generate different contact between subtypes are marked in bold.

B

	1	2	3	4	5	6	7	8	9	10	11	12	13	14	15	16	17
Subtype F V3 loop aminoacid frequency	C (100)	T (100)	R (100)	P(100)	N (100)<br>N (6.25)<br>P(6.25)	N (100)	N (100)	T (100)	R (100)	K (100)	G (6.25)<br>S (93.75)	I (100)	Q (62.5)<br>H (12.5)<br>R (12.5)<br>N (6.25)<br>P(6.25)	I (62.5)<br>L (31.25)<br>M (6.25)	G (100)	P(100)	G (100)
Subtype F V3 loop-CCR5 interactions			R - Y15	P- Y15	N - Y14	N - E18		T- Q188<br>T - Y14	R - P183<br>R - Y184<br>R - Q188	K - Q188<br>K - K191<br>K - E262<br>K - F264		I- S179<br>I - S180	Q - Q170<br>R/H - Q172<br>H/N/Q/R - T177	I/L - W86<br>I/L/M - Y89<br>L - W94<br>I/L/M - T177<br>L/M - C178<br>I/L/M - S179	G - Y89	P- K26<br>P- Y89<br>P- Q280	G - W86<br>G - Y89
	**1**	**2**	**3**	**4**	**5**	**6**	**7**	**8**	**9**	**10**	**11**	**12**	**13**	**14**	**15**	**16**	**17**
Subtype B V3 Loop aminoacid frequency	C (100)	T (86.67)<br>I (8.33)<br>L (1.67)<br>M (1.67)<br>A (1.67)	R (95)<br>G (3.33)<br>K (1.67)	P (98.33)<br>Y (1.67)	N (83.33)<br>S (8.33)<br>T (3.33)<br>G (3.33)<br>H (1.67)	N (98.33)<br>T (1.67)	N (98.33)<br>S (1.67)	T (100)	R (93.33)<br>A (1.67)<br>I (1.67)<br>S (1.67)<br>V (1.67)	K (85)<br>R (10)<br>T (3.33)<br>Q (1.67)	S (70)<br>G (30)	I (98.33)<br>V (1.67)	H (65)<br>N (11.67)<br>P (11.67)<br>S (8.33)<br>T (1.67)<br>Q (1.67)	I (75)<br>M (16.67)<br>L (6.67)<br>V (1.67)	G (96.67)<br>A (3.33)	P (98.33)<br>W (1.67)	G (100)
Subtype B V3 loop-CCR5 Interactions			R/K - Y15	P - Y15	N/S/T - Y14<br>T-Y15	T - Y14<br>N/T - E18	**N**/S - Y14	T - Q188<br>T - Y14	R/I - P183<br>R/I/V - Y184<br>R/I/V - Q188	R/Q/K/T - Q188<br>K/T - K191<br>R - Q261<br>R/Q/K - E262<br>Q/K - F264		I - S179<br>I - S180	Q/T - Q170<br>H - E172<br>N/Q/H - T177<br>**H** - S179	I/L - W86<br>I/L/M/V - Y89<br>L - W94<br>I/L/M - T177<br>L/M - C178<br>I/L/M - S179	A/G - Y89	P/W - K26<br>W - A30<br>P/W - Y89<br>W - A90<br>P- Q280	G - W86<br>G - Y89

	18	19	20	21	22	23	24	25	26	27	28	29	30	31	32	33	34	35
Subtype F V3 loop aminoacid frequency	R (68.75)<br>Q (18.75)<br>K (12.5)	A (87.5)<br>S (12.5)	F (93.75)<br>I (6.25)	Y (100)	A (62.5)<br>T (37.5)	T (100)	G (93.75)<br>E (6.25)	D (62.5)<br>E (12.5)<br>S (12.5)<br>K (6.25)<br>Q (6.25)	I (100)	I (100)	G (100)	D (100)	I (100)	R (100)	K (100)	A (100)	H (93.75)<br>Y (6.25)	C (100)
Subtype F V3 loop-CCR5 interactions	Q/R/K - W86<br>R - Y108<br>Q/R/K - Y251<br>Q/K - E283	A/S - M279	F - Y251<br>F - V254<br>F/I - L255<br>F - N258<br>F/I - M279	Y - N24<br>Y - V25<br>Y - D276<br>Y - M279	A/T - K22	T - Q21<br>T- K22	E - E18<br>E- C20<br>G/E- Q21	Q - Q21<br>K/E - E172		I - M1		D - Y3	I - Y3	R - Y3<br>R - Q4<br>R -D11	K - D11<br>K - I12		H/Y - I12	
	**18**	**19**	**20**	**21**	**22**	**23**	**24**	**25**	**26**	**27**	**28**	**29**	**30**	**31**	**32**	**33**	**34**	**35**
Subtype B V3 Loop aminoacid frequency	R (75)<br>K (13.33)<br>G (6.67)<br>S (3.33)<br>Q (1.67)	A (86.67)<br>T (8.33)<br>V (5)	F (80)<br>L (6.67)<br>M (3.33)<br>V (3.33)<br>W (3.33)<br>Y (1.67)<br>I (1.67)	Y (96.67)<br>F (3.33)	A (65)<br>T (33.33)<br>V (1.67)	T (95)<br>A (3.33)<br>H (1.67)	G (96.67)<br>T (1.67)<br>E (1.67)	D (41.67)<br>E (36.67)<br>Q (8.33)<br>A (5)<br>R (3.33)<br>G (1.67)<br>K (1.67)<br>S (1.67)	I (95)<br>V (5)	I (86.67)<br>V (10)<br>T (3.33)	G (98.33)<br>E (1.67)	D (75)<br>N (23.33)<br>K (1.67)	I (100)	R (96.67)<br>G (1.67)<br>K (1.67)	Q (85)<br>K (11.67)<br>R (3.33)	A (100)	H (85)<br>Y (13.33)<br>Q (1.67)	C (100)
Subtype B V3 loop-CCR5 Interactions	R/Q/G/K/S - W86<br>R- Y108<br>R/Q/K/S - Y251<br>S - M279<br>**R**/Q/K - E283	T/V -Y251<br>A/T/V - M279<br>T/V - Q280	L/M/F/W - Y251<br>F - V254<br>**I**/L/M/F/W/Y – L255<br>F/W - N258<br>L - L275<br>I/L/M/F/W/Y/V - M279	F/Y - K22<br>F/Y - N24<br>F/Y - V25<br>F/Y - D276<br>F/Y - M279<br>F - Q280	A/T/V - K22	T - Q21<br>A/H/T - K22<br>H - E172	E - E18<br>E - C20<br>E/G/T - Q21	R/Q - Q21<br>E/K - E172		I/T/V - M1		K - M1<br>N/D/K - Y3	I - Y3	R - Y3<br>R - Q4<br>R - D11	K/**Q**/R - D11<br>K - I12		Q/H/Y - I12	

Shaded amino acids did not show contacts. Amino acids that were found to generate different contact between subtypes are marked in bold.

Supplementary table 2: table including all datasets used in the work.

Sequence	Dataset
CTRPNNNTVKGIQIGPGRAFLSTGRIIGDIRKAHC	X4-F
CTRPNNNTRKSIQLGPGRAFYATGEIIGDIRKAHC	X4-F
CTRPNNNTRKSIQLGPGRAFYTTGQIIGDIRKAYC	X4-F
CTRPNNNTRKSIRIGPGRAFYTTGAIIGDIRKAHC	X4-F
CTRPNNNTRKSIWIGPGRAFYTTGAIIGDIRKAHC	X4-F
CTRPSNNTSKSIHIGPGRAFHAIGRITGDIRKAHC	X4-F
CTRPSNNTRKAIRIGPGKVVYATGRIIGDIRRAHC	X4-F
CTRPNNNTRKSIQLGPGRAFYTTGQIIGNIRKAYC	X4-F
CTRPNNNTRKSISLGPGRAFYTTGDIIGDIRKAHC	X4-F
CTRPNNNTRKSIQLGPGRAFYATGDIIGDIRQAYC	X4-F
CTRPNNNTRKSIQLGPGRAFYATGDIIGDIRRAHC	X4-F
CTRPNNNTRKGIHIGPGQAFYTTGEIIGDIRKAHC	X4-F
CTRPNNNTRKRIQLGPGHSFHTTGKIIGDIRKAHC	X4-F
CTRPSNNRRKRIHIGPGRAFYTTGGIKGDIRKAYC	X4-F
CTRPNNNTRKRIWIGPGRAFYTGKGIVGDIRKAHC	X4-F
CTRPNNNTRKRIQIGPGRAVYTTGKIIGDIRRAHC	X4-F
CTRPNNNTRKSIYIGPGHAFHTTGRIIGDIRKAHC	X4-F
CTRPNNNTRKSIRIGPGRAVYATGRIIGDIRKAHC	X4-F
CTRPNNNTRKGIHIGPGRAFYATEKIIGDIRKAHC	X4-F
CTRPNNNTVKGIQIGPGRAFRTTGKIIGDIRKAHC	X4-F
CTRPNNNTRKSIHLGPGRAFYTNAEIIGDIRKAHC	X4-F
CTRPNNNTRKGIAIGPGRTFYATEKIIGDIRKAHC	X4-F
CTRPNNNTSTRISIGPGRAFRTMGRIVGDIRMAHC	X4-F
CTRPNNNTRKSIRFGPGQAFYTTGEIIGDIRKAHC	X4-F
CTRPNNNTRKGIHIGPGRAFYATEKIIGDIRKAHC	R5-F
CTRPNNNTRKSIPIGPGKTFYATGDIIGDIRQAHC	R5-F
CTRPNNNTRKSIQLGPGRAFYATGDIIGDIRKAHC	R5-F
CTRPNNNTRKSIQIGPGRAFYTTGDIIGDIRKAHC	R5-F
CTRPNNNTRKSIQIGPGRAFYATGSIIGDIRKAHC	R5-F
CTRPNNNTRKSINIGPGRAFYATGDIIGDIRKAHC	R5-F
CTRPNNNTRKSIRIGPGQSFYTTGDIIGDIRKAHC	R5-F
CTRPNNNTRKSIQLGPGRAIYATGDIIGDIRKAHC	R5-F
CARPNNNTRKSIQIGPGRAFYTTGEIIGDIRKAHC	R5-F
CTRPNNNTRKSIQLGPGKAFYTTGDIIGDIRKAHC	R5-F
CTRPNNNTRKSIQLGPGRAFYTTGEIIGDIRKAHC	R5-F
CTRPNNNTRKSIQMGPGKAFYATGEIIGDIRKAHC	R5-F
CTRPNNNTRKSIHIGPGQAFYATGSIIGDIRKAHC	R5-F
CTRPNNNTRKSIQIGPGRAFYTTGQIIGDIRKAYC	R5-F
CTRPNNNTRKSIPIGPGRAFYATGDIIGDIRKAHC	R5-F
CTRPNNNTRKSIQIGPGRAFYATGDIIGDIRKAHC	R5-F
CTRPNNNTRKSISIGPGRAFYAHGDIVGDIRQAHC	R5-B
CTRYNNNTRKSIHLGPGRAFYATGDIIGDIRQAQC	R5-B
CTRPNNNTRKSIHIAPGRTFYATGDIIGDIRQAHC	R5-B
CTRPNNNTRKSIHMGPGSVWYATGEIIGDIRQAHC	R5-B
CTRPNNNTRKSISIGPGRAFYATGDIIGDIRQAHC	R5-B
CTGPNNNTRKSIHIGPGRAFYTTGEIIGDIRQAHC	R5-B

CTRPNTNTRKSINIGPGRAFYTTGEIIGNIRQAHC	R5-B
CTRPNNNTRKGIHMGPGKVFYATGQIIGNIRQAHC	R5-B
CMRPNNNTRKSIPIGPGRAFYTTGEIIGDIRQAHC	R5-B
CTRPGNNTRKSIPIGPGRAFYATGDIIGDIRQAHC	R5-B
CTRPNNNTRRGIQMGPGRAVYATGDIIGDIRQAHC	R5-B
CTRPNNNTRKGIHIGPGGAIYATGAIIEDIRQAHC	R5-B
CTRPNNNTVKSIHIGPGRAFYTTGQIIGNIRQAHC	R5-B
CTRPNNNTRKSIHMGPGKTFFATGDIIGDIRQAHC	R5-B
CTRPNNNTRKSIHIGPGRAFYTTGEIIGDIRKAHC	R5-B
CTRPNNNTRKGIHIGPGRALYATGDIIGKIRQAHC	R5-B
CLRPNNNTRKGIHIGPGRAFYTTGEIIGDIRQAHC	R5-B
CTRPSNNTRKSINIGPGRAFYTTGEIIGDIRQAHC	R5-B
CIRPNNNTRKSIPMGPGKAFYATGSIIGNIRQAHC	R5-B
CTRPNNNTRKGIHIGPGRAFYATGDIVGDIKQAHC	R5-B
CARPNNNTRKSIHIGPGRAFYTTGEIIGDIRQAHC	R5-B
CTGPNNNTRRSISIGPGGAFYTTGDIIGDIRQAYC	R5-B
CTRPNNNTRKGIHIGPGRAFYTTGEIIGDIRQAHC	R5-B
CTRPTNNTRKSIHIGPGRAFYATGDIIGNIRQAHC	R5-B
CTRPNNNTRKGIHIGPGRAFYTTGEIIGNIRQAHC	R5-B
CTRPNNNTRKGIPIGPGKAFYATGEIIGDIRQAHC	R5-B
CTRPNNNTRKGIHIGPGRAFYTTGGIIGDIRQAHC	R5-B
CTRPSNNTRKSIHMGPGRAFYVTGDVIGDIRQAHC	R5-B
CTRPNNNTRKGIHIGPGKTFYATGQIIGDIRQAHC	R5-B
CTRPNNNTRKSINIGPGRAFFATGDIIGDIRQAHC	R5-B
CTRPNNSTRKSIHMGWGRAFYATGEIIGNIRQAHC	R5-B
CTRPNNNTRKSISIGPGRAFYATGEIIGDIRQAYC	R5-B
CIRPNNNTRKSIHMGPGRAFYATGDVIGDIRKAYC	R5-B
CTRPNNNTIKGIHIGPGRAFYTTGQVIGDIRKAYC	R5-B
CTRPNNNTRKSINIGPGGAFYAATDIIGDIRQAHC	R5-B
CTRPTNNTRKGIHIGPGRAFYTTGEIIGDIRKAHC	R5-B
CTRPNNNTAKGIHIGPGRAMYATERIVGNIRRAHC	R5-B
CTKPNNNTRRSISLGPGRAYYATGDIIGDIRQAHC	R5-B
CTRPNNNTRKSIHIGPGRAFYTTGDIIGDIRKAHC	R5-B
CTRPNNNTRRSIHIGPGSAFYATGDIIGDIRQAHC	R5-B
CTRPNNNTRKSIHIGPGRAFYATGEIVGNIRQAHC	R5-B
CTRPNNNTRKSINIGPGRAWYATGKIIGNIRQAHC	R5-B
CTRPNNNTRRSIHIGPGRAFYTTGEIVGDIRQAHC	R5-B
CTRPNNNTRKGIHIGPGRTFYATGEIVGDIRQAHC	R5-B
CTRPGNNTRKSINIGPGRALYTTGDIIGDIRQAHC	R5-B
CTRPNNNTRKSIHIAPGRAFYATGDIIGDIRQAYC	R5-B
CIRPSNNTRTSIHLGPGQAVYATGEIIGNIRQAHC	R5-B
CIRPNNNTRKGVHLGPGGALYATGAIIGDIRQAYC	R5-B
CTRPNNNTRTGIPMGPGRAMYATGDIIGNIRQAYC	R5-B
CTRPNNNTRKGIHIGPGRVFYATGEIIGDIRQAHC	R5-B
CIRPNNNTRKSINIGPGRAFYTTGAITGDIRQAHC	R5-B
CTRPHNNTRKSIHIGPGRAFYATGQITGDIRQAYC	R5-B
CTRPNNNTRKSIHIGPGRAFYTTGEIIGNIRQAHC	R5-B
CTRPSNNTRKSITIGPGKAFYATGEIIGDIRKAHC	R5-B

CTRPNNNTRKSIHIGPGRAFYTAGEIIGDIRQAHC	R5-B
CTRPNNNTRKSIHVGPGKTLYATGDIIGDIGQAHC	R5-B
CTRPNNNTRRSIPIGPGRAFYATGDIIGDIRKAHC	R5-B
CTRPNNNTRKSIHMGPGKAFYATGDIIGDIRRAHC	R5-B
CTRPSNNTRKSIPIGPGRAFYATGDIIGNIRQAHC	R5-B
CTRPNNNTSQSIHIGPGRAFYATGRIIGDIRQAHC	R5-B
CTRPNNNIRKRIHIGPGRAFYTTRQIIGNIRQAHC	X4-B
CTRPNNNVRKRISLSPGRVYYTTGEIRGDIRKAYC	X4-B
CTRPNKNIRKGLRLGPGRAFYTMGGIVGYIRQAHC	X4-B
CTRPNNNTRRRIYIGQGRAVYTTKQIVGDIRKAYC	X4-B
CARPNNNTRKGIHMGPGRAMYATEKITGDIRQAHC	X4-B
CTRPNKTTRKGLRLGPGRAFYTMGGIVGYIRQAHC	X4-B
CARPNNNTRKRIYMGTGRYMSATEKITGDIRQARC	X4-B
CTRPNNNTRKRIYIGQGRAVYTTKQIVGDIRKAYC	X4-B
CTRPNKNIRKGLRLGPGRAFYTMGGIVGNIRQTHC	X4-B
CTRPNKTIRKGLRLGPGRAFYTMGGIEGYIRQAHC	X4-B
CTRPNKTTRKGLRLGPGRAFYTLGGIVGYIRQAHC	X4-B
CTRPNNNTRKRIYIGQGRAVYTTRQIIGDIRKAYC	X4-B
CTRPNKTIRKGLRLGPGRAFYTMGRIEGYIRQAHC	X4-B
CTRPGNKTIRSISLGPGRAFSATRQIIGDIRKAYC	X4-B
CTRPNNNTRKRITAGPGRVLYTTGQIIGDIRRAHC	X4-B
CTRPNNNTRNRIYIGQGRAVYTTKQIIGDIRKAYC	X4-B
CTRPGNKTIRSISLGPGRAFSVTRQIIGDIRKAYC	X4-B
CTRPYNYKKKKITTGPGRVLYTTEEIIGDIRRAHC	X4-B
CTRPNYNKRKRIHIGPGRAFYTTKNIIGTIRQAHC	X4-B
CTRPNNNTRNRIYIGQGRAVYTTKQIVGDIRKAYC	X4-B
CTRPNNNTRKRIYIGQGRAVYTTRQIVGDIRKAYC	X4-B
CTRPGNNTRRRISIGPGRAFYTTEQIIGNIRQAHC	X4-B
CTRPNNYKKKRITTGPGRVLYTTGQIIGDIRRAYC	X4-B
CTRPNNYKKKRITVGPGRVLYTTGQIIGDIRRAHC	X4-B
CTRPNNYKKKRITIGPGRVLYTTGQIIGDIRRAYC	X4-B
CTRPNNYKRKRITTGPGRVLYTTGQIIGDIRRAYC	X4-B
CTRPNNHTRKRVTLGPSRVYYTTGEITGDIRRAHC	X4-B
CTRPNNNTRKGIHIGLGRRVYVTRQIIGDTKRAHC	X4-B
CTRPNNNTRQRISIGPGRAFYTTRQVVGDIRQAHC	X4-B
CTRPNNNTRKRISIGPGRAFYTTRQVIGDIRQAHC	X4-B
CTRPNNNTRNRISIGPGRAFYTTRQVIGDIRQAHC	X4-B
CTRPSNNTRRRISIGPGRAFYTTRQVIGDIRQAHC	X4-B
CTRPNNNTRRRLSIGPGRAFYATRDIIGDIRQAHC	X4-B
CTRPNNNTRGRLSIGPGRAFYATRDIIGDIRRAHC	X4-B
CTRPNNNTMKSITIGPGRAFYTTGQIIGDIRQAHC	X4-B
CTRPNNNTRKRVSIGPGRAWYTTKQIVGDIRQAHC	X4-B
CTRPNNNTRKRVTLGPGRVWYTTGQIIGDIRKAHC	X4-B
CIRPNNNTRKSIPLGPGKAWYTTGEIIGDIRKAHC	X4-B
CTRPNNNTKKGIYVGPGRKVYTTDRIIGDIRQAHC	X4-B
CTRPNNNTKRGIYVGPGRKVYTTDRIIGDIRQAHC	X4-B
CTRPNNNTKRGIYVGPGRKVYTTDRIIGNIRQAHC	X4-B
CTRPFKNVRTSLRIGPGRVFYRTGGITGDIRKAYC	X4-B

Supplementary table 3: 5-fold cross-validation results at different False Positive Rate values for Geno2pheno, $WebPSSM_{sinsi}$, 11,22,25 X4 Energy model and mixed model.

	FPR 10%		FPR 15%		FPR 20%	
Method	Sensitivity	Specificity	Sensitivity	Specificity	Sensitivity	Specificity
Mixed model W=0.3	0.60	0.89	0.68	0.89	0.68	0.89
WebPSSM	0.44	0.94	0.44	0.89	0.44	0.89
Geno2Pheno	0.56	0.94	0.64	0.86	0.68	0.83
11,22,25 X4 Energy	0.36	0.72	0.40	0.72	0.40	0.72

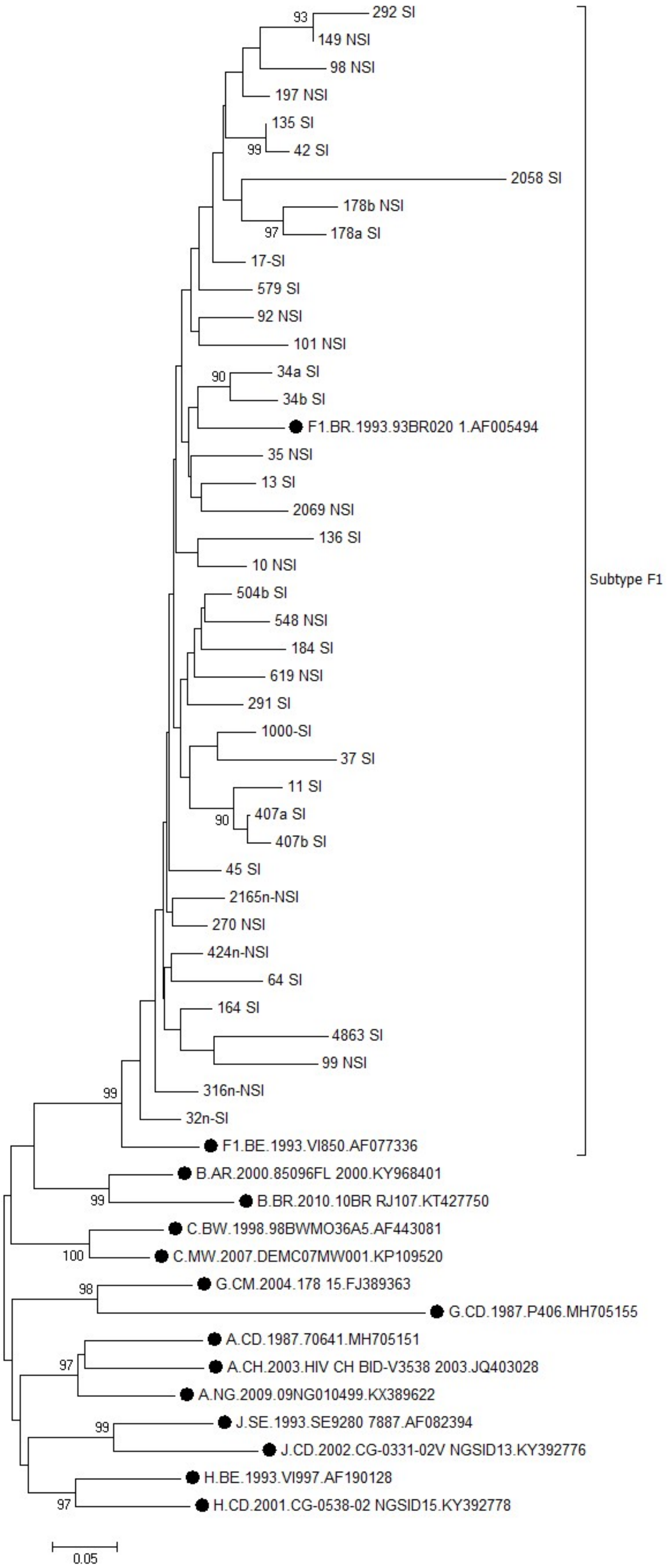

Supplementary figure 1: Neighbor-Joining phylogenetic tree of HIV-1 env nucleotide sequences spanning positions 7089 to 7250 of HXB2 reference genome. Bootstrap support above 90% are shown at branch nodes. Subtype reference genomes retrieved from GenBank are shown with filled circles.
